# Blood pressure parameters and cognitive decline and dementia after stroke or transient ischemic attack: results from the PROGRESS trial

**DOI:** 10.1093/ajh/hpag056

**Published:** 2026-06-19

**Authors:** Sultana Shajahan, Mark Woodward, Craig S Anderson, Cheryl Carcel, Linan Chen, Ruth Peters, Karin Rådholm, Stephen Harrap, John Chalmers, Katie Harris

**Affiliations:** The George Institute for Global Health, The University of New South Wales, Sydney, Australia; The George Institute for Global Health, The University of New South Wales, Sydney, Australia; The George Institute for Global Health, Imperial College London, London, United Kingdom; The George Institute for Global Health, The University of New South Wales, Sydney, Australia; Institute of Science and Technology for Brain-inspired Intelligence, Fudan University, Shanghai, China; Neurology Department, Royal Prince Alfred Hospital, Sydney, Australia; The George Institute for Global Health, The University of New South Wales, Sydney, Australia; Prince of Wales Clinical School, The University of New South Wales, Sydney, Australia; The George Institute for Global Health, The University of New South Wales, Sydney, Australia; The George Institute for Global Health, The University of New South Wales, Sydney, Australia; The George Institute for Global Health, The University of New South Wales, Sydney, Australia; Department of Health, Medicine, and Caring Sciences, Linköping University, Linköping, Sweden; Primary Health Care Centre Kärna, Region Östergötland, Linköping, Sweden; Department of Anatomy and Physiology, University of Melbourne and Royal Melbourne Hospital, Parkville, Australia; The George Institute for Global Health, The University of New South Wales, Sydney, Australia; The George Institute for Global Health, The University of New South Wales, Sydney, Australia

**Keywords:** blood pressure, cognitive decline, dementia, stroke, pulse pressure

## Abstract

**Background:**

Besides elevated mean systolic blood pressure (BP), BP variability, pulse pressure (PP), or mean arterial pressure (MAP) could be important targets for dementia prevention. This study investigated these associations in participants with stroke or transient ischemic attack from the Perindopril Protection Against Recurrent Stroke Study (PROGRESS) trial.

**Methods:**

BP parameters were calculated from measurements for 18 months on six occasions. Risk of cognitive decline according to Mini-Mental State Examination scores and/or adjudicated dementia outcomes per 1-standard deviation higher for each BP parameter was calculated using modified Poisson regression models with a log link. Subgroup analysis was conducted by mild cognitive impairment (MCI) at baseline, sex, and stroke subtype.

**Results:**

Of 5128 participants recruited between May 1995 and November 1997, 567 (11.1%) developed cognitive decline/dementia after 4 (median) years of follow-up until 2001. For systolic BP (SBP), higher mean, maximum, cumulative load, and last measure were associated with higher risk of cognitive decline/dementia, but mean conferred the highest risk (relative risk 1.18, 95% confidence intervals [CIs] 1.06-1.31). For diastolic BP, higher mean, maximum, variability, cumulative load, slope, and last measure were associated, while load showed the strongest association (1.23, 95% CI 1.12-1.36). Only the mean PP was associated (1.11, 95% CI 1.00-1.24). For MAP, MAP load showed the strongest association (1.20, 95% CI 1.09-1.33). Systolic, diastolic, and MAP variability showed a higher risk in those without MCI.

**Conclusions:**

Besides mean SBP, higher variability, cumulative load, slope, and last measures of diastolic BP, PP, and MAP were associated with increased risk of cognitive decline/dementia.

**Clinical Trial Registration:**

This trial was not registered because enrollment began before July 1, 2005.

## Graphical abstract

**Graphical Abstract:**
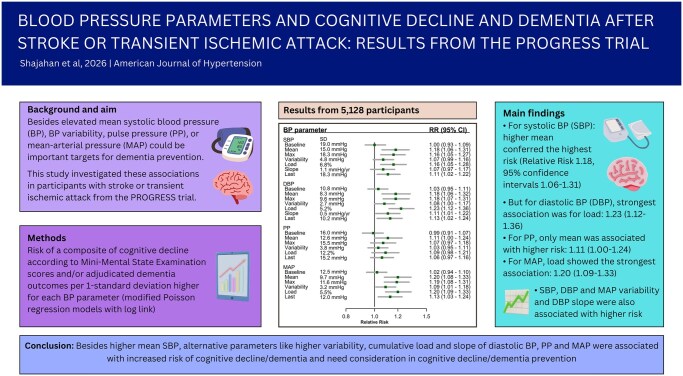

## Introduction

A history of stroke or transient ischemic attack (TIA) is associated with an elevated risk of dementia and cognitive impairment.^1^ High blood pressure (BP) is a key modifiable risk factor for the prevention of both dementia and stroke.^2^ Aside from elevated mean systolic BP (SBP), other hemodynamic measures could also be important as additional targets for dementia prevention.^3^ Blood pressure variability (BPV), independent of mean BP, predicts subclinical target organ damage and cardiovascular disease.^3^^,^^4^ Higher BPV has been previously found to be associated with increased risk of stroke and cerebral small vessel disease.^5^^,^^6^ As stroke impairs cerebral regulation, even small fluctuations in BP could cause under- or over-perfusion in the affected areas.^5^^,^^7^ After ischemic stroke, a sudden drop in BP can reduce the probability of reperfusion of the affected tissue, while high BP can risk hemorrhagic transformation of the affected area.^8^ In hemorrhagic stroke, BPV is known to aggravate the hematoma expansion and worsen functional outcome.^5^^,^^9^ Thus, further research is needed to understand the risk of higher BPV with dementia and cognitive decline in those with a history of stroke/TIA.

Other BP parameters may also provide additional insights. For example, widening pulse pressure (PP) is a marker of vascular aging and arterial stiffening, which is considered to be a high predictor of cardiovascular disease risk.^10^ Mean arterial pressure (MAP) is another potentially important parameter, defined as the average arterial pressure throughout a cardiac cycle of systole and diastole. Higher MAP levels are related to cardiovascular disease and target organ damage, whereas low levels may be detrimental in hemodynamically unstable, critically ill patients.^11^ Similarly, BP load has emerged as an additional parameter to predict risk of cardiovascular disease by calculating the area under the curve of several BP measures.^12^ However, the associations of the aforementioned measures on cognitive outcomes in those with a history of cerebrovascular disease remain unclear. Most prior work links baseline BP to incident dementia,^13^^,^^14^ but the impact of BP change over time in people with stroke/TIA is unknown, despite one third of ischemic stroke survivors developing cognitive impairment.^15^ It is also unclear whether BP-cognition associations are stronger in those with mild cognitive impairment (MCI). The aims of this study were to determine the relationship between clinically important BP parameters and cognitive decline and/or dementia in those with a history of stroke/TIA, and whether these associations differ by MCI at baseline.

## Methods

### Study population

This study included participants from the Perindopril Protection Against Recurrent Stroke Study (PROGRESS), the details of which are described in detail elsewhere.^16–18^ In brief, PROGRESS was a multicenter, multinational, randomized, placebo-controlled trial with a flexible approach to BP-lowering in 6105 participants from 172 centers in Asia, Australasia, and Europe between May 1995 and November 1997. Participants were eligible if they had a stroke due to cerebral ischemia, intracerebral hemorrhage, or TIA within the previous 5 years. The active treatment consisted of perindopril (4 mg/day) for all participants, with additional indapamide (2.5 mg/day) in patients for whom the responsible physician judged there to be no specific indication for, or contraindication to, treatment with a diuretic. The primary outcome was total stroke (fatal or non-fatal). Potentially eligible individuals entered a 4-week pre-randomization run-in period in which they received open-label perindopril. The mean follow-up duration was 3.9 years. The institutional ethics committee of each collaborating center approved the trial, and all participants provided written informed consent.

### BP parameters

In the year after randomization, participants were seen on five occasions. In the second and subsequent years, visits were held every 6 months. At these visits, the data on BP and cognitive function were collected. BP was measured in duplicate, to the nearest 2 mm Hg, with a standard mercury sphygmomanometer. For the present analysis, participants with repeated BP measurements on six occasions were included during an 18-month exposure window, at months 1, 3, 6, 9, 12, and 18 months post-randomization. BP readings at randomization were excluded to allow for stabilization of treatment effects initiated at randomization. A timeline of the measurement occasions of the exposure is provided in Figure S1, and details of all included parameters and their calculations are provided in Table S1.

### Assessment of cognitive function and dementia outcomes

The primary outcome in the present study was a composite of cognitive decline and/or dementia. Individuals with a prior or current diagnosis of dementia at baseline (ie, at registration) were excluded. Cognitive function was assessed using the Mini-Mental State Examination (MMSE),^19^ at baseline, at 6 and 12-month visits, and annually until the end of follow-up.^17^ Cognitive decline was defined as a drop of 3 points or more between the baseline and last recorded MMSE scores.^17^ Both dementia and cognitive function were prespecified secondary outcomes of the PROGRESS trial.^18^^,^^20^ A two-person Dementia Adjudication Committee reviewed the clinical assessment and collected information to confirm or refute the diagnosis of dementia by consensus agreement. Further details on dementia screening and diagnosis are provided in Appendix S1.

### Cognitive impairment at baseline

Participants were categorized by their cognition levels at baseline. In this study, a score <28 on the MMSE was considered as MCI, and 28-30 as having an absence of MCI.^19^^,^^21^ Those without baseline MMSE scores were excluded.

### Statistical analysis

Baseline characteristics by MCI vs an absence of MCI at baseline were summarized as the mean and standard deviation (SD) for continuous variables, numbers with percentages for categorical variables, and medians with interquartile range for variables with non-normal distributions. Differences between groups were calculated using the Chi‑square test for categorical variables, Welch’s *t*-test for continuous variables and the Wilcoxon-Mann-Whitney test for non-normally distributed variables. Summary statistics were calculated for each BP parameter. Modified Poisson regression models with log link and robust (sandwich) standard errors were used to estimate relative risks (RRs) with 95% confidence intervals (CIs) per 1-SD higher of (a) baseline BP measures, (b) calculated BP parameters, and (c) last BP measures, in separate models with a composite outcome of cognitive decline/dementia. Three sets of models were fit, (1) univariate, and adjusted for baseline covariates: (2) basic models adjusted for age, sex, age at completion of highest education, region of residence, baseline BP measure, and randomized BP treatment allocation; and (3) multiple adjusted models further adjusted for MMSE at baseline, body mass index, estimated glomerular filtration rate (eGFR), smoking, current drinking status (yes/no), and diabetes. SBP parameters were adjusted for baseline SBP, similarly, diastolic blood pressure (DBP) parameters for baseline DBP, and so on. Since there were no covariates with >5% missing (Table S2), it was not necessary to conduct multiple imputation, and a complete case analysis was done.^22^ Multiple adjusted restricted cubic splines were used to explore non-linear shapes of the associations between each parameter and cognitive decline/dementia with 4 knots (at the 5th, 35th, 65th, and 95th percentiles).^23^ Subgroup analyses were conducted by the presence of MCI measured by MMSE at baseline (vs an absence of MCI), randomized BP treatment allocation (active vs placebo), sex (women vs men), stroke subtype/TIA (ischemic vs hemorrhagic vs TIA), and history of cerebrovascular disease (>1 event or not). A test for interaction was used to assess any differences in the risk of cognitive decline/dementia between groups, and a sensitivity analysis was conducted using the coefficient of variation (CV) as an alternative measure for BP variability, calculated using the formula (SD/mean) × 100. A further sensitivity analysis was conducted by adjusting the BP variability analysis by mean BP levels. Since the onset of dementia is prodromal and death can occur before the onset of dementia, multinomial logistic regression models were conducted to account for the competing risk of death, which were compared to binomial logistic regression models.^24^ A *P-*value <.05 was considered statistically significant. All analyses were conducted using R Studio version 4.3.0.

## Results

A total of 5128 participants with six BP and ≥2 MMSE measurements were included in this analysis (Figure S2), including 12.8% with MCI at baseline (Table 1). At baseline, in comparison to the group with an absence of MCI, more women had MCI, and those with MCI were more likely to be hypertensive, have lower eGFR, higher percentage of diabetes, and a history of ischemic or hemorrhagic stroke with more than one cerebrovascular event; conversely, this group were less likely to be from Asia, consume alcohol, and have a previous TIA (Table 1).

**Table 1:** Baseline characteristics of PROGRESS participants by presence of mild cognitive impairment (MCI) at baseline.

Characteristics	**MCI** ^a^ ** (*n* = 657)**	Absence of MCI (*n* = 4471)	Total (*n* = 5128)	*P*-value
Age, years (SD)	66.9 (8.5)	63.5 (9.4)	64 (9.4)	<.01
Female, *n* (%)	240 (36.5)	1308 (29.3)	1548 (30.2)	<.001
Active treatment, *n* (%)	334 (50.8)	2221 (49.7)	2555 (49.8)	.61
Asia, *n* (%)	233 (35.5)	1887 (42.2)	2120 (41.3)	<.01
Mean age at completion of highest education, years (SD)	15.9 (5.6)	18.0 (6.3)	17.7 (6.2)	<.01
MMSE score, median (interquartile interval)	24 (22-25)	29 (28-30)	29 (27-30)	<.01
Blood pressure				
Mean SBP, mm Hg (SD)	146.3 (18.9)	149.1 (19.7)	146.6 (19.0)	<.01
Mean DBP, mm Hg (SD)	85.2 (11.2)	85.7 (10.7)	85.6 (10.8)	.33
Hypertensive, *n* (%)	480 (73.1)	3073 (68.7)	3553 (69.3)	.12
BMI, kg/m^2^, mean (SD)	25.6 (4.1)	25.7 (3.7)	25.7 (3.7)	.31
eGFR <60 mL/min/1.73 m^2^, *n* (%)	140 (21.3)	679 (15.2)	819 (16.0)	<.01
Ever smoker, *n* (%)	374 (56.9)	2578 (57.7)	2952 (57.6)	.74
Current drinker, *n* (%)	229 (34.9)	1805 (40.4)	2034 (39.7)	.01
History of concurrent disease				
Diabetes, *n* (%)	100 (15.2)	514 (11.5)	614 (12.0)	.01
Myocardial infarction, *n* (%)	46 (7.0)	294 (6.6)	340 (6.6)	.74
Atrial fibrillation, *n* (%)	58 (8.8)	318 (7.1)	376 (7.3)	.13
Peripheral arterial disease, *n* (%)	37 (5.6)	157 (3.5)	194 (3.8)	.01
Types of previous cerebrovascular events				
TIA/amaurosis fugax, *n* (%)	79 (12)	780 (17.4)	859 (16.8)	.01
Ischemic stroke only, *n* (%)	458 (69.7)	3044 (68.1)	3502 (68.3)	.13
Hemorrhagic stroke only, *n* (%)	86 (13.1)	456 (10.2)	542 (10.6)	.03
Ischemic or hemorrhagic stroke, *n* (%)	544 (82.8)	3500 (78.3)	4044 (78.9)	<.01
Unknown stroke, *n* (%)	34 (5.2)	191 (4.3)	225 (4.4)	<.01
>1 previous cerebrovascular event, *n* (%)	150 (22.8)	888 (19.9)	1038 (20.2)	.09
Outcomes				
Cognitive decline/dementia, *n* (%)	181 (27.5)	386 (8.6)	567 (11.1)	<.01
Cognitive decline only, *n* (%)	98 (14.9)	367 (8.2)	465 (9.1)	<.01
Dementia only, *n* (%)	138 (21.0)	123 (2.8)	261 (5.1)	<.01
Cognitive decline and dementia,^b^ *n* (%)	55 (8.4)	104 (2.3)	159 (3.1)	<.01
Death, *n* (%)	57 (8.7)	246 (5.5)	303 (5.9)	<.01

Abbreviations: BMI, body mass index; DBP, diastolic blood pressure; eGFR, estimated glomerular filtration rate; SBP, systolic blood pressure; SD, standard deviation; TIA, transient ischemic attack.

a Mild cognitive impairment (MCI) at baseline was measured using the Mini-Mental State Examination (MMSE) score.

b Participants who had both cognitive decline and dementia.

After a median of 4 years of follow-up, in those with MCI at baseline, 181 (27.5%) of participants developed cognitive decline/dementia, whereas 386 (8.6%) developed the outcome in the group without MCI at baseline There was a total of 57 (8.7%) deaths recorded in those with MCI, compared to 246 (5.5%) in the group having an absence of MCI (Table 1).

### BP parameters and cognitive decline/dementia

Participants had a mean baseline SBP of 146.6 (SD 19) mm Hg and DBP of 85.6 (10.8) mm Hg, and a mean SBP of 137.7 (15) mm Hg and a mean DBP of 81 (8.3) mm Hg, in the 18-month exposure window (Table S3).

**Figure 1:**
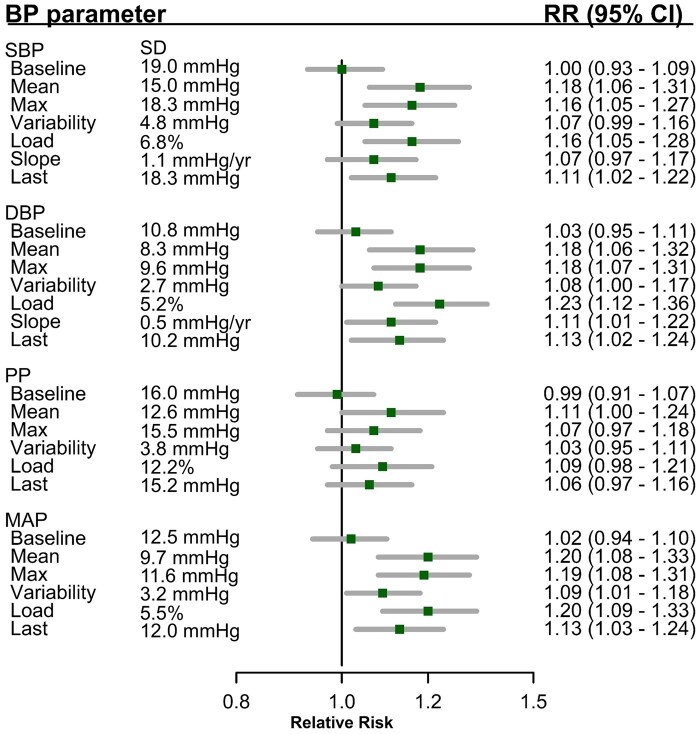
Multiple adjusted relative risks (RRs) per standard deviation (SD) higher with 95% confidence intervals (CIs) for cognitive decline/dementia for each blood pressure (BP) parameter. Note: BP parameters were calculated separately in each multiple regression model. The results were adjusted for age, sex, age at completion of highest education, region of residence, baseline BP measure, randomized BP treatment allocation, Mini-Mental State Examination (MMSE) at baseline, body mass index (BMI), estimated glomerular filtration rate (eGFR), smoking, current drinking status (yes/no), and diabetes. SBP, systolic blood pressure; DBP, diastolic blood pressure; PP, pulse pressure; MAP, mean arterial pressure.

**Figure 2:**
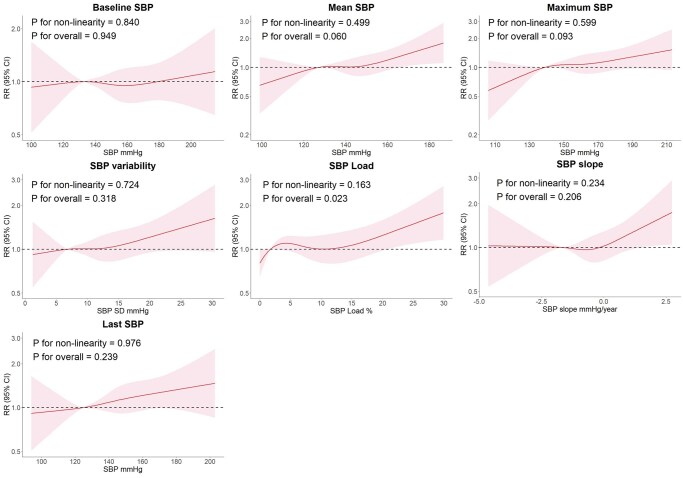
Splines of each systolic blood pressure (SBP) parameter with the outcomes of cognitive decline/dementia. *Note*: The results were adjusted for age, sex, age at completion of highest education, region of residence, baseline blood pressure (BP) measure, randomized BP treatment allocation, Mini-Mental State Examination (MMSE) at baseline, body mass index (BMI), estimated glomerular filtration rate (eGFR), smoking, current drinking ­status (yes/no), and diabetes. RR, relative risk; CI, confidence interval; SD, standard deviation.

For SBP, 1-SD higher mean (RR 1.18, 95% CI 1.06-1.31), maximum measure (1.16, 1.05-1.27), and cumulative load (1.16, 1.05-1.28) were associated with cognitive decline/dementia in the multiple adjusted models (Figure 1 and Table S4). SBP variability had a borderline association in the multiple adjusted model (RR 1.07, 95% CI 0.99-1.16). The SBP slope was no longer associated with cognitive decline/dementia in the multiple-adjusted model. The slope displayed a J-shaped association with cognitive outcomes, as shown in Figure 2.

For DBP, the strongest association for higher risk of cognitive decline/dementia was found for 1-SD higher cumulative load (RR 1.23, 95% CI 1.12-1.36), followed by the mean (1.18, 1.06-1.32), and the maximum measure (1.18, 1.07-1.31), after multiple adjustments were made for covariates (Figure 1). 1-SD higher DBP variability was also associated with the outcome (RR 1.08, 95% CI 1.00-1.17). Higher DBP slope remained associated with cognitive decline/dementia after multiple adjustments (RR 1.11, 95% CI 1.01-1.22) and displayed a J-shaped association with cognitive outcomes, as shown in Figure S3.

The only measure of PP associated with cognitive decline/dementia was the mean PP (RR 1.11, 95% CI 1.00-1.24; Figure 1).

For MAP, 1-SD higher cumulative load and mean showed the strongest associations (RR 1.20, 95% CI 1.09-1.33 and RR 1.20, 95% CI 1.08-1.33, respectively; Figure 1). MAP variability was also associated (RR 1.09, 95% CI 1.01-1.18).

Except for PP, the last measures of all the parameters were associated with cognitive decline/dementia: SBP, RR 1.11 (95% CI 1.02-1.22); DBP 1.13 (1.02-1.24), and MAP 1.13 (1.03-1.24); Figure 1.

Multiple adjusted restricted cubic spline analyses showed a non-linear association for mean DBP (p for non-linearity = 0.01; Figure S3), but not for the rest of the parameters (Figure 2 and Supplementary Figures S4 and S5).

### Results from subgroup and sensitivity analysis

There were interactions between those with MCI vs an absence of MCI at baseline for associations of SBP, DBP, and MAP variability with cognitive decline/dementia (p for interaction ≤0.01; Table 2). No interactions were found by other subgroups (Tables S5-S9). In sensitivity analysis, CV was a comparable measure of variability for all BP parameters to SD in multiple adjusted models (Table S10). After adjusting for mean BP levels, the association for SBP variability was slightly attenuated from RR 1.07 (95% CI 0.99-1.16) to RR 1.05 (95% CI 0.97-1.14), while DBP and MAP variability remained borderline associated with cognitive decline/dementia: 1.07 (0.99-1.16) and 1.08 (0.99-1.16), respectively (Table S11). The results were similar for all parameters when comparing results from binomial logistic regression to multinomial models (Table S12).

**Table 2:** Relative risks (RRs) for the associations of blood pressure (BP) parameters per standard deviation higher and cognitive decline/dementia by presence of mild cognitive impairment (MCI) at baseline.

BP parameter	Standard deviation	With MCI RR (95% CI)	Absence of MCI RR (95% CI)	Test for interaction
SBP				
Baseline SBP	19.0 mm Hg	0.94 (0.83-1.07)	1.03 (0.93-1.15)	0.32
Mean SBP	15.0 mm Hg	1.13 (0.97-1.32)	1.18 (1.03-1.35)	0.72
Max SBP	18.3 mm Hg	1.06 (0.92-1.22)	1.18 (1.04-1.33)	0.34
SBP variability	4.8 mm Hg	0.92 (0.80-1.04)	1.13 (1.03-1.24)	0.02
SBP load	6.8%	1.09 (0.94-1.26)	1.18 (1.04-1.34)	0.45
SBP slope	1.1 mm Hg/yr	1.01 (0.89-1.14)	1.09 (0.96-1.23)	0.45
Last SBP	18.3 mm Hg	1.09 (0.96-1.24)	1.11 (0.99-1.25)	0.84
DBP				
Baseline DBP	10.8 mm Hg	0.99 (0.87-1.11)	1.06 (0.96-1.18)	0.42
Mean DBP	8.3 mm Hg	1.15 (0.97-1.36)	1.16 (1.01-1.34)	0.90
Max DBP	9.6 mm Hg	1.06 (0.90-1.24)	1.21 (1.07-1.37)	0.26
DBP variability	2.7 mm Hg	0.89 (0.77-1.03)	1.16 (1.06-1.27)	0.01
DBP load	5.2%	1.18 (1.02-1.37)	1.25 (1.11-1.41)	0.36
DBP slope	0.5 mm Hg/yr	1.11 (0.95-1.29)	1.11 (0.99-1.25)	0.95
Last DBP	10.2 mm Hg	1.16 (0.99-1.36)	1.12 (0.99-1.26)	0.73
Pulse pressure				
Baseline PP	16.0 mm Hg	0.94 (0.82-1.07)	1.00 (0.90-1.11)	0.50
Mean PP	12.6 mm Hg	1.08 (0.92-1.27)	1.12 (0.98-1.28)	0.78
Max PP	15.5 mm Hg	0.97 (0.82-1.15)	1.11 (0.98-1.26)	0.28
PP variability	3.8 mm Hg	0.92 (0.80-1.05)	1.07 (0.97-1.17)	0.11
PP load	12.2%	1.03 (0.87-1.21)	1.12 (0.98-1.28)	0.48
Last PP	15.2 mm Hg	1.03 (0.90-1.18)	1.06 (0.95-1.19)	0.78
MAP				
Baseline MAP	12.5 mm Hg	0.96 (0.85-1.08)	1.05 (0.95-1.16)	0.31
Mean MAP	9.7 mm Hg	1.15 (0.98-1.35)	1.19 (1.04-1.37)	0.75
Max MAP	11.6 mm Hg	1.06 (0.91-1.23)	1.22 (1.08-1.38)	0.21
MAP variability	3.2 mm Hg	0.90 (0.78-1.02)	1.18 (1.07-1.29)	<0.01
MAP load	5.5%	1.14 (0.99-1.31)	1.23 (1.09-1.39)	0.47
Last MAP	12.0 mm Hg	1.13 (0.98-1.29)	1.13 (1.00-1.27)	0.99

Relative risks (RRs) were reported per standard deviation higher with 95% confidence intervals (CIs) for cognitive decline/dementia for different blood pressure (BP) parameters in those with mild cognitive impairment (MCI) vs. an absence of MCI at baseline. *P-*value for the test for interaction was presented in the right-hand column. Models were adjusted for baseline BP, age, sex, education, region, randomized BP treatment allocation, body mass index (BMI), estimated glomerular filtration rate (eGFR), smoking, current drinker or not, and diabetes.

Abbreviations: DBP, diastolic blood pressure; MAP, mean arterial pressure; PP, pulse pressure; SBP, systolic blood pressure.

## Discussion

In this analysis of PROGRESS involving patients with a history of stroke/TIA, higher BP summary measures, including the mean, maximum, variability, and load of SBP and DBP, were all significantly associated with future cognitive decline/dementia. In addition, the DBP slope, mean PP, the mean, maximum value, variability, and load of MAP, and the last BP measures, were also associated with poor cognitive outcomes. Furthermore, higher mean DBP showed a significant non-linear association with cognitive decline/dementia. When stratified by the presence of MCI at baseline (measured by MMSE), SBP, DBP, and MAP variability, all showed a higher risk of poor cognitive outcomes in the group with an absence of MCI at baseline.

In our study, higher mean and maximum SBP and DBP were associated with cognitive decline/dementia in those with previous stroke/TIA, which is in line with previous studies examining the link between hypertension and later life dementia,^25^^,^^26^ or post-stroke cognitive impairment.^27^ Constant elevations in BP can induce adaptive changes to reduce mechanical stress on the arterial wall, such as hypertrophic remodeling, accumulation of extracellular matrix proteins in the vessel wall, decreased elasticity of vessel walls, and impaired cerebral autoregulation, causing brain tissue damage.^28^ Further neuropathological mechanisms may be associated with post-stroke cognitive decline, although these are complex and incompletely understood.^15^ However, there are immediate effects of tissue damage from vessel occlusion (ischemia) or rupture (hemorrhage), which may lead to oxidative stress, blood-brain barrier dysfunction, inflammation, and neuronal death.^15^ Microvascular damage and blood-brain barrier leakage may cause further vasogenic edema and microhemorrhages in the affected tissues.^15^ The evidence on post-stroke cognitive impairment is contradictory. In a cross-sectional analysis of 432 dementia-free subjects, no associations were found between SBP, DBP, PP, and MAP with cognition, each measured in the 90-day period after stroke.^29^ In contrast, our findings show that after 4 years of median follow-up, SBP, DBP, and MAP were each associated with poor outcomes. The time after a stroke could be a key factor when assessing the subsequent risk of post-stroke cognitive impairment or dementia.

In our study, SBP, DBP, and MAP variability were associated with cognitive decline/dementia, which supports previous evidence where mid-term BPV in the early phase of acute ischemic stroke was independently associated with post-stroke cognitive impairment.^30^ Several mechanisms may explain the consequences of higher BPV following cerebral infarction or hemorrhage.^5^ With impaired cerebral autoregulation after a stroke, small fluctuations in BP may lead to under- or over-perfusion.^7^ Sudden drops in BP reduce the chance of reperfusion of potentially viable ischemic tissue and increase the risk of further ischemic expansion of the lesion.^8^ In hemorrhagic stroke, wide BP fluctuations during the active bleeding stage can worsen hematoma growth, while recurrent drops in BP can lead to secondary tissue injury.^9^

Higher mean PP was associated with poorer cognitive outcomes. High PP in older people (age ≥75 years) is a marker of increased large artery stiffness and widespread atherosclerosis.^31^ An analysis of the Framingham Heart Study showed that, along with increasing age, the contributions of DBP to SBP in predicting chronic heart disease progressively diminished, and that from PP increased.^32^ Higher mean PP could also be a predictor of dementia in older adults with previous stroke/TIA.

The association of MAP and post-stroke cognitive outcomes has been somewhat overlooked. Higher mean, maximum, load, and MAP variability were associated with poor cognitive outcomes in post-stroke/TIA patients. A study examining 3-month functional outcomes in stroke patients undergoing mechanical thrombectomy revealed that a drop in MAP < 60 mm Hg favored lower modified Rankin scale scores (3-6).^33^ The commonly recommended minimal threshold for adequate cerebral blood flow autoregulation is a MAP ≥60 mm Hg in the absence of increased intracranial pressure.^33^ From a pathophysiological perspective, MAP is an important and commonly used BP target value indicating adequate cerebral blood flow.^33^ Identifying which thresholds of MAP lead to better post-stroke cognitive outcomes could be beneficial for future practice.

In our study, higher cumulative SBP, DBP, and MAP loads were associated with poorer cognitive outcomes. Previously, cumulative BP load was also associated with the risk of cognitive decline and dementia in cognitively healthy adults aged ≥50 years.^34^ Recent evidence suggests that due to BP fluctuations, reliance on a single BP measurement does not accurately predict cardiovascular outcomes.^12^ Recently, there has been growing interest in using cumulative BP load exposure as a supplementary parameter, which considers the time attribute of BP fluctuations,^12^ which in turn could potentially enhance the accuracy of risk models compared to conventional baseline BP assessment.^12^

Last measures of SBP, DBP, PP, and MAP were all associated with cognitive decline/dementia, but not the baseline measures. This implies that BP measures closer to the time of the outcome may be more clinically relevant than baseline measures. Moreover, J-shaped relationships were found between both SBP and DBP slopes and cognitive decline/dementia. Previous evidence supports the dynamic rise of SBP/DBP over time to be associated with worse cognitive ­outcomes.^35^ Slopes reflecting the dynamic change of BP over time could more accurately inform future risk of cognitive decline/dementia compared to a single baseline BP measurement.

Mean DBP showed a significant non-linear association with cognitive decline/dementia in our analysis. Previous evidence found a non-linear relationship between BP levels and MCI progression, demonstrating pre-hypertensive levels to be the critical BP management threshold for MCI onset.^36^ This has implications for the treatment of high BP in patients with non-linear BP associations, emphasizing the importance of not only lowering high BP but also monitoring and potentially treating low BP to preserve cognitive function.

We found that, when stratified by MCI at baseline, SBP, DBP, and MAP variability showed a higher risk of poorer cognitive outcomes in the group with an absence of MCI at baseline. Conversely, in the analysis of the Systolic Blood Pressure Intervention Trial Memory and Cognition in Decreased Hypertension (SPRINT MIND) dataset, the authors found that increased BPV after MCI diagnosis was associated with incident probable dementia.^37^ The lack of associations between some BP parameters and cognitive outcomes in the MCI group may reflect more advanced structural or neurovascular pathology, such that the effect of BP is attenuated once substantial organic brain damage is already present.

Our study had several strengths. First, it included a large representative sample of participants with prior cerebrovascular disease, investigated several clinically important measures of BP, and the analyses were adjusted for multiple confounders, including cardiovascular risk factors and baseline BP. Since BP data were collected for research purposes, the SBP and DBP measurements are more likely to be standardized. All cases were reviewed by a central Dementia Adjudication Committee to ensure accurate dementia diagnosis. Finally, multinomial models were conducted to account for the competing risk of death. However, this study also had some limitations. Cognitive decline was measured using MMSE, which lacks sensitivity in detecting MCI.^38^ As this was a secondary analysis of a trial dataset, there may be potential selection bias. Since BP measures in the PROGRESS trial may have been measured at different times of the day, the measures of variability in this study may simply reflect normal fluctuations throughout the day.^39^ Despite adjusting for important demographic and cardiovascular covariates in our final analysis, the possibility of residual confounding cannot be fully excluded.^40^ Finally, PP and MAP are both mathematically associated with SBP and DBP, and their results are likely to be correlated. This may complicate efforts to attribute variable-specific underlying pathological mechanisms. Multiple testing of interactions raises the concern of false positives. With the threshold of 5%, one test in 20 would be expected to be significant even if there were no real effects. Thus, our results should be interpreted with caution. Some important directions for future research in this area are provided in the Supplementary discussion section.

In conclusion, in addition to higher mean SBP, alternative measures such as variability, cumulative load, slope, and last measures of DBP, PP, and MAP were associated with cognitive decline/dementia. This highlights the need to consider additional BP parameters in risk assessments to guide clinical management and prevent further cognitive decline.

## Supplementary Material

hpag056_Supplementary_Data

## Data Availability

The data underlying this article cannot be shared publicly for the privacy of individuals who participated in the study and were used by the agreement of the PROGRESS Management Committee for the current study.

## References

[1] Pendlebury ST , Rothwell PM. Prevalence, incidence, and factors associated with pre-stroke and post-stroke dementia: a systematic review and meta-analysis. Lancet Neurol. 2009;8:1006-1018. 19782001 10.1016/S1474-4422(09)70236-4

[2] Peters R , Xu Y, Fitzgerald O, et al; Dementia rIsk REduCTion (DIRECT) Collaboration. Blood pressure lowering and prevention of dementia: an individual patient data meta-analysis. Eur Heart J. 2022;43:4980-4990. 36282295 10.1093/eurheartj/ehac584

[3] Ohkuma T , Woodward M, Jun M, et al; ADVANCE Collaborative Group. Prognostic value of variability in systolic blood pressure related to vascular events and premature death in type 2 diabetes mellitus. Hypertension. 2017;70:461-468. 28584014 10.1161/HYPERTENSIONAHA.117.09359

[4] Iadecola C , Gottesman RF. Neurovascular and cognitive dysfunction in hypertension: epidemiology, pathobiology, and treatment. Circ Res. 2019;124:1025-1044. 30920929 10.1161/CIRCRESAHA.118.313260 PMC6527115

[5] Lattanzi S , Brigo F, Silvestrini M. Blood pressure variability and stroke: a risk marker of outcome and target for intervention. J Clin Hypertens (Greenwich). 2021;23:103-105. 33125836 10.1111/jch.14092 PMC8029899

[6] Tully PJ , Yano Y, Launer LJ, et al; Variability in Blood Pressure and Brain Health Consortium. Association between blood pressure variability and cerebral small‐vessel disease: a systematic review and meta‐analysis. J Am Heart Assoc. 2020;9:e013841. 31870233 10.1161/JAHA.119.013841 PMC6988154

[7] Stead LG , Gilmore RM, Vedula KC, Weaver AL, Decker WW, Brown RD. Impact of acute blood pressure variability on ischemic stroke outcome. Neurology. 2006;66:1878-1881. 16801654 10.1212/01.wnl.0000219628.78513.b5

[8] Zhang T , Wang X, Wen C, et al Effect of short-term blood pressure variability on functional outcome after intra-arterial treatment in acute stroke patients with large-vessel occlusion. BMC Neurol. 2019;19:228. 31558167 10.1186/s12883-019-1457-5 PMC6764143

[9] Rodriguez-Luna D , Piñeiro S, Rubiera M, et al Impact of blood pressure changes and course on hematoma growth in acute intracerebral hemorrhage. Eur J Neurol. 2013;20:1277-1283. 23647568 10.1111/ene.12180

[10] Melgarejo JD , Thijs L, Wei DM, et al Relative and absolute risk to guide the management of pulse pressure, an age-related cardiovascular risk factor. Am J Hypertens. 2021;34:929-938. 33687055 10.1093/ajh/hpab048 PMC8457427

[11] Papaioannou TG , Protogerou AD, Vrachatis D, et al Mean arterial pressure values calculated using seven different methods and their associations with target organ deterioration in a single-center study of 1878 individuals. Hypertens Res. 2016;39:640-647. 27194570 10.1038/hr.2016.41

[12] You FF , Zhong WF, Gao YN, et al Cumulative blood pressure predicts risk of cardiovascular outcomes in middle-aged and older population. Ann Med. 2025;57:2476735. 40066575 10.1080/07853890.2025.2476735 PMC11899200

[13] Lennon MJ , Makkar SR, Crawford JD, Sachdev PS. Midlife hypertension and Alzheimer’s disease: a systematic review and meta-analysis. J Alzheimers Dis. 2019;71:307-316. 31381518 10.3233/JAD-190474

[14] Ou YN , Tan CC, Shen XN, et al Blood pressure and risks of cognitive impairment and dementia: a systematic review and meta-analysis of 209 prospective studies. Hypertension. 2020;76:217-225. 32450739 10.1161/HYPERTENSIONAHA.120.14993

[15] Kalaria RN , Akinyemi R, Ihara M. Stroke injury, cognitive impair­ment and vascular dementia. Biochim Biophys Acta. 2016;1862:915-925. 26806700 10.1016/j.bbadis.2016.01.015 PMC4827373

[16] PROGRESS Collaborative Group. Randomised trial of a perindopril-based blood-pressure-lowering regimen among 6,105 individuals with previous stroke or transient ischaemic attack. Lancet. 2001;358:1033-1041. 11589932 10.1016/S0140-6736(01)06178-5

[17] Tzourio C , Anderson C, Chapman N, et al Effects of blood pressure lowering with perindopril and indapamide therapy on dementia and cognitive decline in patients with cerebrovascular disease. Arch Intern Med. 2003;163:1069-1075. 12742805 10.1001/archinte.163.9.1069

[18] PROGRESS Management Committee. Blood pressure lowering for the secondary prevention of stroke: rationale and design for PROGRESS. J Hypertens Suppl. 1996;14:S41-5. discussion S5–6. 8934377

[19] Crum RM , Anthony JC, Bassett SS, Folstein MF. Population-based norms for the Mini-Mental State Examination by age and educational level. JAMA. 1993;269:2386-2391. 8479064

[20] Tzourio C , Anderson C. Blood pressure reduction and risk of dementia in patients with stroke: rationale of the dementia assessment in PROGRESS (perindopril protection against recurrent stroke study). PROGRESS management committee. J Hypertens Suppl. 2000;18:S21-4. 10939786

[21] de Galan BE , Zoungas S, Chalmers J, et al; ADVANCE Collaborative Group. Cognitive function and risks of cardiovascular disease and hypoglycaemia in patients with type 2 diabetes: the action in diabetes and vascular disease: preterax and diamicron modified release controlled evaluation (ADVANCE) trial. Diabetologia. 2009;52:2328-2336., 19688336 10.1007/s00125-009-1484-7

[22] Woodward M. Epidemiology: Study Design and Data Analysis. 3rd ed. CRC Press; 2014.

[23] Durrleman S , Simon R. Flexible regression models with cubic splines. Stat Med. 1989;8:551-561. 2657958 10.1002/sim.4780080504

[24] Gong J , Harris K, Tzourio C, et al Sex differences in predictors for cognitive decline and dementia in people with stroke or transient ischemic attack in the PROGRESS trial. Int J Stroke. 2022;17:863-872. 34791978 10.1177/17474930211059298

[25] Lennon MJ , Lam BCP, Lipnicki DM, et al Use of antihypertensives, blood pressure, and estimated risk of dementia in late life: an individual participant data meta-analysis. JAMA Netw Open. 2023;6:e2333353. 37698858 10.1001/jamanetworkopen.2023.33353 PMC10498335

[26] Whitmer RA , Sidney S, Selby J, Johnston SC, Yaffe K. Midlife cardiovascular risk factors and risk of dementia in late life. Neurology. 2005;64:277-281. 15668425 10.1212/01.WNL.0000149519.47454.F2

[27] Huang H , Zhan Y, Yu L, Li S, Cai X. Association between blood pressure and post-stroke cognitive impairment: a meta-analysis. Rev Cardiovasc Med. 2024;25:174. 39076476 10.31083/j.rcm2505174 PMC11267189

[28] Lee KP , Chang AYW, Sung PS. Association between blood pressure, blood pressure variability, and post-stroke cognitive impairment. Biomedicines. 2021;9:773. 34356837 10.3390/biomedicines9070773 PMC8301473

[29] Levine DA , Galecki AT, Okullo D, et al Association of blood pressure and cognition after stroke. J Stroke Cerebrovasc Dis. 2020;29:104754. 32370925 10.1016/j.jstrokecerebrovasdis.2020.104754 PMC7934908

[30] Geng S , Liu N, Meng P, et al Midterm blood pressure variability is associated with poststroke cognitive impairment: a prospective cohort study. Front Neurol. 2017;8:365. 28804475 10.3389/fneur.2017.00365 PMC5532726

[31] Safar ME. Pulse pressure in essential hypertension: clinical and therapeutical implications. J Hypertens. 1989;7:769-776. 2685115 10.1097/00004872-198910000-00001

[32] Franklin SS , Larson MG, Khan SA, et al Does the relation of blood pressure to coronary heart disease risk change with aging? The Framingham Heart Study. Circulation. 2001;103:1245-1249. 11238268 10.1161/01.cir.103.9.1245

[33] Fandler-Höfler S , Heschl S, Argüelles-Delgado P, et al Single mean arterial blood pressure drops during stroke thrombectomy under general anaesthesia are associated with poor outcome. J Neurol. 2020;267:1331-1339. 31955244 10.1007/s00415-020-09701-x PMC7184049

[34] Li C , Zhu Y, Ma Y, Hua R, Zhong B, Xie W. Association of cumulative blood pressure with cognitive decline, dementia, and mortality. JACC. J Am Coll Cardiol. 2022;79:1321-1335. 35393012 10.1016/j.jacc.2022.01.045

[35] Zhu Y , Li C, Gao D, et al Associations of blood pressure trajectories with subsequent cognitive decline, dementia and mortality. J Prev Alzheimers Dis. 2024;11:1426-1434. 39350390 10.14283/jpad.2024.91

[36] Yi F , Gao Y, Liu X, et al A non-linear relationship between blood pressure and mild cognitive impairment in elderly individuals: a cohort study based on the Chinese Longitudinal Healthy Longevity Survey (CLHLS). Neurol Sci. 2024;45:4817-4828. 38676817 10.1007/s10072-024-07539-z

[37] de Havenon A , Muddasani V, Anadani M, Prabhakaran S. Impact of mean blood pressure and blood pressure variability after diagnosis of mild cognitive impairment and risk of dementia. J Clin Hypertens (Greenwich). 2021;23:2124-2128. 34862714 10.1111/jch.14391 PMC8696239

[38] Mitchell AJ. The Mini-Mental State Examination (MMSE): update on its diagnostic accuracy and clinical utility for cognitive disorders. In: Larner AJ, ed. Cognitive Screening Instruments: A Practical Approach. Springer International Publishing; 2017:37-48.

[39] Mancia G , Ferrari A, Gregorini L, et al Blood pressure and heart rate variabilities in normotensive and hypertensive human beings. Circ Res. 1983;53:96-104. 6861300 10.1161/01.res.53.1.96

[40] Cheng X , Song C, Ouyang F, et al Systolic blood pressure variability: risk of cardiovascular events, chronic kidney disease, dementia, and death. Eur Heart J. 2025;46:2673-2687. 40249367 10.1093/eurheartj/ehaf256 PMC12257294

