# Supplementary material for: Blood pressure parameters and cognitive decline and dementia after stroke or transient ischemic attack: results from the PROGRESS trial
Source: Am J Hypertens. 2026 Jun 19;39(9):1151–9. doi: 10.1093/ajh/hpag056 (PMC13473881; doi:10.1093/ajh/hpag056)
Supplement: Supplementary file 1 [file hpag056_supplementary_data.docx]

**Supplementary Methods**

**Appendix S1. Screening and diagnosis of dementia**

Missing Mini-Mental State Examination (MMSE) values at baseline were imputed as the values recorded at the 6-month visit, when possible, for n = 7. After imputing, it yielded 6,080 individuals with baseline MMSE, 25 were not imputed. The remaining 25 participants were excluded from all analyses, as were 192 participants with a single MMSE score recorded throughout the study.

During the study follow-up, a 2-phase screening assessment was used for the diagnosis of dementia.^1^ Participants were screened positive for ‘possible dementia’, and whether they satisfied any of the following criteria: (i) MMSE score of ≤25 at any follow-up visit; (ii) a decline in MMSE score of ≥3 points between any two follow-up visits; (iii) MMSE scores missing for ≥2 scheduled follow-up visits; (iv) a positive response by the investigator to the question, “In your opinion, does this patient have dementia?” Screen-positive participants were referred to a local specialist experienced in dementia for diagnosis. The clinical assessment included an interview with the patient and a close friend or relative. If the patient could not be assessed or died, then information was collected through other sources, including medical records, and interviews with family members and/or other medical practitioners, using a checklist based on the Diagnostic and Statistical Manual of Mental Disorders, Fourth Edition (DSM-IV).^2^

**Supplementary Tables**

**Supplementary Table S1: Definitions of blood pressure parameters included in the main analysis**

| **BP parameter** | **Definition** |
| --- | --- |
| PP | Difference between the SBP and DBP |
| MAP | Calculated using the formula [DBP]+0.412 × [PP] |
| Baseline SBP, DBP, PP and MAP | First measures at baseline |
| Mean SBP, DBP, PP and MAP | Average over the exposure period |
| Maximum SBP, DBP, PP and MAP | Maximum value for the exposure period |
| Variability (SD) of SBP, DBP, PP and MAP | Standard deviation of BP measures over the exposure period |
| Cumulative SBP, DBP, PP and MAP load | Area under the curve (AUC) for SBP values ≥130 mmHg and DBP ≥80 mmHg, divided by the AUC for all measured SBP and DBP values, respectively, and then multiplied by 100  The cut offs for SBP and DBP were used to calculate cumulative loads for PP and MAP |
| SBP and DBP slope | Calculated with the BP at registration and at least one other BP measurement during the exposure period at 1, 3, 6, 9, 12 and 18 months, using linear mixed models with a random intercept and slope for individuals |
| Last SBP, DBP, PP and MAP | Last measures at 18 months |

Note: BP summary parameters calculated using the six BP measurements were the mean, maximum, variability, cumulative load, and slope of SBP and DBP, and the mean, maximum, variability and load of PP and MAP, where PP = SBP – DBP, and MAP = [DBP]+0.412 × [PP].^3^ Visit-to-visit variability was defined using the standard deviation (SD) of BP measurements. Cumulative SBP and DBP loads were calculated as the AUC for SBP values ≥130 mmHg and DBP ≥80 mmHg, divided by the AUC for all measured SBP and DBP values, respectively, and then multiplied by 100 using a formula published previously.^4^ AUC was calculated by connecting all points in a direct line using the composite trapezoid rule. The cut-offs for SBP and DBP values were used to calculate the PP and MAP loads. The slopes for SBP and DBP were calculated with the BP at registration and at least one other BP measurement during the exposure period, at 1, 3, 6, 9, 12 and 18 months.^5,6^ The slopes for SBP and DBP were calculated using linear mixed models with a random intercept and slope for individuals.^5,6^ PP=Pulse Pressure, SBP=Systolic Blood Pressure, DBP=Diastolic Blood Pressure, MAP=Mean Arterial Pressure.

**Supplementary Table S2: Number of participants with missing covariate data**

| **Covariate** | **Number of participants with missing data in study sample, n= 5,128** |
| --- | --- |
| Age | 0 |
| Sex | 0 |
| Age at completion of highest education | 182 (3.5%) |
| Region of residence | 0 |
| MMSE at baseline | 0 |
| Body Mass Index | 20 (0.4%) |
| Estimated Glomerular Filtration Rate (eGFR) <60 mL/min/1.73 m^2^ | 75 (1.5%) |
| Ever smoker | 3 (0.1%) |
| Current drinker | 3 (0.1%) |
| Concurrent diabetes | 0 |

Note: MMSE= Mini-Mental State Examination

**Supplementary Table S3: Summary statistics of BP parameters**

| **BP Parameter** | **Min** | **Median** | **Mean** | **Max** | **SD** | **IQI** |
| --- | --- | --- | --- | --- | --- | --- |
| **SBP** |  |  |  |  |  |  |
| Baseline SBP, mmHg | 90 | 146 | 146.6 | 245 | 19 | 133 – 160 |
| Mean SBP, mmHg | 87.5 | 137.2 | 137.7 | 204.2 | 15 | 127.3 – 147.5 |
| Max SBP, mmHg | 90 | 150 | 151.6 | 242 | 18.3 | 139 – 162.5 |
| SBP variability, SD, mmHg | 0.0 | 9.5 | 10.2 | 37.9 | 4.8 | 6.8 – 12.9 |
| SBP load, % | 0.0 | 6.3 | 7.7 | 37.2 | 6.8 | 1.7 – 12 |
| SBP variability, CV % | 0.0 | 7 | 7.4 | 25.2 | 3.3 | 5 – 9.3 |
| SBP Slope, mmHg/year | -6 | -1 | -1 | 4 | 1.1 | -1.7 – (-0.3) |
| Last SBP, mmHg | 81 | 137 | 138 | 242 | 18.3 | 125 – 150 |
| **DBP** |  |  |  |  |  |  |
| Baseline DBP, mmHg | 50 | 85 | 85.6 | 131 | 10.8 | 79 – 92 |
| Mean DBP, mmHg | 48.9 | 81 | 81 | 119 | 8.3 | 75.5-86.3 |
| Max DBP, mmHg | 53.5 | 89 | 89 | 139 | 9.6 | 82-95 |
| DBP variability, SD, mmHg | 0.0 | 5.7 | 6.1 | 20.4 | 2.7 | 4.2-7.6 |
| DBP load, % | 0.0 | 3.3 | 5.0 | 33.6 | 5.2 | 0.5-8 |
| DBP variability, CV % | 0.0 | 7.1 | 7.6 | 24.4 | 3.3 | 5.2-9.6 |
| DBP Slope, mmHg/year | -2.6 | -0.6 | -0.6 | 1.7 | 0.5 | -0.9 – (-0.3) |
| Last DBP, mmHg | 40.00 | 80.00 | 80.74 | 129.00 | 10.2 | 74.00 – 88.00 |
| **PP** |  |  |  |  |  |  |
| Baseline PP, mmHg | 21 | 60 | 61 | 155 | 16 | 50 – 70.5 |
| Mean PP, mmHg | 21.3 | 55.8 | 56.8 | 108.8 | 12.6 | 47.7 – 64.5 |
| Max PP, mmHg | 24.5 | 66.5 | 67.8 | 142 | 15.5 | 57 – 77.1 |
| PP variability, SD, mmHg | 0.6 | 7.7 | 8.2 | 37 | 3.8 | 5.5 – 10.3 |
| PP load, % | 0.0 | 12.4 | 14.4 | 55.2 | 12.2 | 3.5 – 23.1 |
| PP variability, CV % | 1.1 | 13.9 | 14.6 | 58.3 | 6.4 | 10 – 18.3 |
| Last PP, mmHg | 16 | 56 | 57.2 | 131 | 15.2 | 47.00 – 66.62 |
| **MAP** |  |  |  |  |  |  |
| Baseline MAP, mmHg | 66.5 | 110.6 | 110.7 | 171.5 | 12.5 | 101.8 – 118.7 |
| Mean MAP, mmHg | 66.4 | 104.2 | 104.4 | 154.1 | 9.7 | 97.8 – 110.8 |
| Max MAP, mmHg | 72.4 | 113.3 | 113.8 | 174.7 | 11.6 | 105.9 – 121.2 |
| MAP variability, SD, mmHg | 0.2 | 6.5 | 7.0 | 25.1 | 3.2 | 4.7 – 8.8 |
| MAP load, % | 0.0 | 4.7 | 6.0 | 35.6 | 5.5 | 1.3 – 9.5 |
| MAP variability, CV % | 0.2 | 6.3 | 6.7 | 22 | 3 | 4.6 – 8.4 |
| Last MAP, mmHg | 60.6 | 103.9 | 104.3 | 174.7 | 12 | 96.2 – 112.1 |

Note: SD=Standard deviation; IQI=Interquartile interval; SBP=Systolic Blood Pressure; CV=Coefficient of variation; DBP=Diastolic Blood Pressure; PP=Pulse Pressure; MAP=Mean Arterial Pressure.

**Supplementary Table S4: Unadjusted and adjusted relative risks (RR) per standard deviation (SD) higher with 95% confidence intervals (CI) for cognitive decline/dementia for different blood pressure (BP) parameters**

| **BP Parameter** | **Univariate**  **RR (95% CI)** | **Basic Adjusted***  **RR (95% CI)** | **Multiple Adjusted** RR (95% CI)** |
| --- | --- | --- | --- |
| **SBP** |  |  |  |
| Baseline SBP | 1.11 (1.03-1.19) | 1.02 (0.94-1.10) | 1.00 (0.93-1.09) |
| Mean SBP | 1.25 (1.16-1.35) | 1.19 (1.07-1.32) | 1.18 (1.06-1.31) |
| Maximum SBP | 1.26 (1.17-1.35) | 1.18 (1.08-1.30) | 1.16 (1.05-1.27) |
| SBP variability (SD) | 1.19 (1.11-1.28) | 1.10 (1.02-1.19) | 1.07 (0.99-1.16) |
| SBP Load | 1.24 (1.16-1.33) | 1.18 (1.07-1.30) | 1.16 (1.05-1.28) |
| SBP Slope | 1.05 (0.96-1.14) | 1.10 (1.00-1.20) | 1.07 (0.97-1.17) |
| Last SBP | 1.18 (1.09-1.27) | 1.13 (1.03-1.23) | 1.11 (1.02-1.22) |
| **DBP** |  |  |  |
| Baseline DBP | 0.93 (0.86-1.01) | 1.04 (0.96-1.13) | 1.03 (0.95-1.11) |
| Mean DBP | 0.99 (0.91-1.07) | 1.17 (1.04-1.31) | 1.18 (1.06-1.32) |
| Maximum DBP | 1.03 (0.94-1.11) | 1.18 (1.07-1.31) | 1.18 (1.07-1.31) |
| DBP variability (SD) | 1.12 (1.04-1.21) | 1.09 (1.01-1.18) | 1.08 (1.00-1.17) |
| DBP Load | 1.05 (0.97-1.13) | 1.26 (1.14-1.39) | 1.23 (1.12-1.36) |
| DBP Slope | 1.08 (0.99-1.17) | 1.14 (1.04-1.25) | 1.11 (1.01-1.22) |
| Last DBP | 1.00 (0.92-1.09) | 1.14 (1.04-1.26) | 1.13 (1.02-1.24) |
| **PP** |  |  |  |
| Baseline PP | 1.18 (1.09-1.27) | 1.00 (0.91-1.08) | 0.99 (0.91-1.07) |
| Mean PP | 1.30 (1.21-1.39) | 1.13 (1.02-1.26) | 1.11 (1.00-1.24) |
| Maximum PP | 1.28 (1.20-1.37) | 1.10 (1.00-1.22) | 1.07 (0.97-1.18) |
| PP variability (SD) | 1.16 (1.08-1.25) | 1.05 (0.97-1.14) | 1.03 (0.95-1.11) |
| PP load | 1.29 (1.21-1.38) | 1.12 (1.01-1.24) | 1.09 (0.98-1.21) |
| Last PP | 1.21 (1.13-1.30) | 1.07 (0.97-1.17) | 1.06 (0.97-1.16) |
| **MAP** |  |  |  |
| Baseline MAP | 1.03 (0.96-1.11) | 1.03 (0.95-1.12) | 1.02 (0.94-1.10) |
| Mean MAP | 1.15 (1.06-1.24) | 1.20 (1.08-1.34) | 1.20 (1.08-1.33) |
| Maximum MAP | 1.17 (1.08-1.26) | 1.20 (1.09-1.33) | 1.19 (1.08-1.31) |
| MAP variability (SD) | 1.17 (1.09-1.26) | 1.11 (1.03-1.20) | 1.09 (1.01-1.18) |
| MAP load | 1.17 (1.09-1.26) | 1.24 (1.12-1.36) | 1.20 (1.09-1.33) |
| Last MAP | 1.11 (1.03-1.21) | 1.15 (1.05-1.26) | 1.13 (1.03-1.24) |

Note: *Basic adjusted Model: Adjusted for baseline BP, age, sex, education, region, and BP treatment allocation group. **Multiple adjusted model: Adjusted for baseline BP, age, sex, education, region, BP treatment allocation group, Mini-Mental State Examination score (MMSE) at baseline, body mass index (BMI), estimated glomerular filtration rate (eGFR), smoking, current drinker or not, and diabetes. BP=Blood pressure; RR=Relative risk; CI=Confidence interval; SBP=Systolic blood pressure; SD=Standard deviation; DBP=Diastolic blood pressure; PP=Pulse pressure; MAP=Mean arterial pressure.

**Supplementary Table S5: Relative risks (RR) per SD higher and 95% confidence intervals (CIs) for cognitive decline/dementia for different blood pressure (BP) parameters by BP treatment allocation group**

| **BP Parameter** | **BP treatment group**  **RR (95% CI)** | **Placebo group**  **RR (95% CI)** | **Test for interaction** |
| --- | --- | --- | --- |
| **SBP** |  |  |  |
| Baseline SBP | 0.98 (0.87-1.11) | 1.03 (0.92-1.15) | 0.62 |
| Mean SBP | 1.19 (1.03-1.37) | 1.17 (1.00-1.36) | 0.89 |
| Max SBP | 1.14 (1.00-1.31) | 1.16 (1.01-1.34 | 0.88 |
| SBP variability (SD) | 1.05 (0.94-1.16) | 1.08 (0.97-1.21) | 0.68 |
| SBP Load | 1.17 (1.01-1.35) | 1.14 (1.00-1.31) | 0.84 |
| SBP Slope | 1.10 (0.98-1.24) | 1.02 (0.90-1.17) | 0.44 |
| Last SBP | 1.12 (0.99-1.26) | 1.10 (0.96-1.25) | 0.83 |
| **DBP** |  |  |  |
| Baseline DBP | 1.00 (0.89-1.12) | 1.05 (0.94-1.18) | 0.55 |
| Mean DBP | 1.20 (1.03-1.41) | 1.16 (1.00-1.35) | 0.75 |
| Max DBP | 1.18 (1.02-1.36) | 1.19 (1.04-1.36) | 0.94 |
| DBP variability (SD) | 1.05 (0.94-1.18) | 1.09 (0.98-1.21) | 0.14 |
| DBP Load | 1.30 (1.12-1.52) | 1.18 (1.04-1.33) | 0.34 |
| DBP Slope | 1.16 (1.01-1.33) | 1.06 (0.93-1.20) | 0.37 |
| Last DBP | 1.14 (0.98-1.32) | 1.11 (0.98-1.26) | 0.81 |
| **PP** |  |  |  |
| Baseline PP | 0.98 (0.87-1.11) | 1.00 (0.89-1.12) | 0.83 |
| Mean PP | 1.11 (0.96-1.29) | 1.11 (0.95-1.29) | 0.98 |
| Max PP | 1.11 (0.96-1.29) | 1.10 (0.95-1.28) | 0.57 |
| PP variability (SD) | 1.02 (0.91-1.13) | 1.04 (0.92-1.16) | 0.83 |
| PP load | 1.11 (0.96-1.28) | 1.07 (0.91-1.25) | 0.74 |
| Last PP | 1.07 (0.95-1.21) | 1.05 (0.92-1.19) | 0.82 |
| **MAP** |  |  |  |
| Baseline MAP | 0.99 (0.88-1.11) | 1.04 (0.94-1.16) | 0.55 |
| Mean MAP | 1.21 (1.04-1.41) | 1.18 (1.02-1.38) | 0.83 |
| Max MAP | 1.18 (1.02-1.36) | 1.19 (1.04-1.36) | 0.92 |
| MAP variability (SD) | 1.06 (0.94-1.18) | 1.11 (1.00-1.23) | 0.07 |
| MAP load | 1.25 (1.08-1.44) | 1.17 (1.03-1.33) | 0.54 |
| Last MAP | 1.14 (1.00-1.30) | 1.12 (0.98-1.27) | 0.84 |

Note: p-value calculated using t-test between the BP parameter and BP treatment group allocation was used to test the significance of the interaction term. The results were adjusted for age, sex, age at completion of highest education, region of residence, baseline BP measure, Mini-Mental State Examination (MMSE) score at baseline, body mass index (BMI), estimated glomerular filtration rate (eGFR), smoking, current drinking status (yes/no), and diabetes. SBP=Systolic blood pressure; SD=Standard deviation; DBP=Diastolic blood pressure; PP=Pulse pressure; MAP=Mean arterial pressure.

**Supplementary Table S6: Relative risks (RR) per SD higher and 95% confidence intervals (CIs) for cognitive decline/dementia for different blood pressure (BP) parameters by sex**

| **BP Parameter** | **Women**  **OR (95% CI)** | **Men**  **OR (95% CI)** | **Test for interaction** |
| --- | --- | --- | --- |
| **SBP** |  |  |  |
| Baseline SBP | 1.03 (0.88-1.20) | 1.00 (0.91-1.10) | 0.77 |
| Mean SBP | 1.22 (1.01-1.47) | 1.16 (1.02-1.31) | 0.68 |
| Max SBP | 1.20 (1.01-1.42) | 1.14 (1.01-1.27) | 0.63 |
| SBP variability (SD) | 1.06 (0.92-1.23) | 1.08 (0.98-1.18) | 0.86 |
| SBP Load | 1.21 (1.02-1.44) | 1.13 (1.00-1.28) | 0.54 |
| SBP Slope | 1.05 (0.88-1.24) | 1.07 (0.97-1.20) | 0.81 |
| Last SBP | 1.13 (0.95-1.34) | 1.11 (1.00-1.23) | 0.82 |
| **DBP** |  |  |  |
| Baseline DBP | 1.01 (0.85-1.20) | 1.04 (0.95-1.13) | 0.78 |
| Mean DBP | 1.23 (1.02-1.48) | 1.16 (1.01-1.33) | 0.62 |
| Max DBP | 1.27 (1.08-1.49) | 1.15 (1.02-1.30) | 0.40 |
| DBP variability (SD) | 1.17 (1.03-1.34) | 1.04 (0.95-1.15) | 0.20 |
| DBP Load | 1.35 (1.15-1.59) | 1.19 (1.05-1.34) | 0.24 |
| DBP Slope | 1.10 (0.93-1.29) | 1.11 (0.99-1.25) | 0.88 |
| Last DBP | 1.10 (0.92-1.31) | 1.14 (1.02-1.28) | 0.71 |
| **PP** |  |  |  |
| Baseline PP | 1.03 (0.89-1.19) | 0.97 (0.88-1.07) | 0.56 |
| Mean PP | 1.12 (0.92-1.35) | 1.11 (0.97-1.26) | 0.94 |
| Max PP | 1.10 (0.91-1.33) | 1.05 (0.94-1.19) | 0.71 |
| PP variability (SD) | 0.99 (0.86-1.15) | 1.05 (0.95-1.15) | 0.58 |
| PP load | 1.09 (0.89-1.33) | 1.09 (0.96-1.24) | 0.99 |
| Last PP | 1.10 (0.93-1.31) | 1.04 (0.94-1.16) | 0.60 |
| **MAP** |  |  |  |
| Baseline MAP | 1.02 (0.87-1.20) | 1.02 (0.93-1.11) | 0.97 |
| Mean MAP | 1.25 (1.04-1.50) | 1.17 (1.03-1.34) | 0.60 |
| Max MAP | 1.21 (1.03-1.43) | 1.18 (1.05-1.33) | 0.81 |
| MAP variability (SD) | 1.13 (0.99-1.30) | 1.08 (0.98-1.18) | 0.60 |
| MAP load | 1.31 (1.12-1.53) | 1.16 (1.03-1.30) | 0.27 |
| Last MAP | 1.13 (0.95-1.35) | 1.14 (1.02-1.27) | 0.97 |

Note: p-value calculated using t-test between the BP parameter and sex was used to test the significance of the interaction term. The results were adjusted for age, age at completion of highest education, region of residence, baseline BP measure, randomized BP treatment allocation, Mini-Mental State Examination (MMSE) score at baseline, body mass index (BMI), estimated glomerular filtration rate (eGFR), smoking, current drinking status (yes/no), and diabetes. SBP=Systolic blood pressure; SD=Standard deviation; DBP=Diastolic blood pressure; PP=Pulse pressure; MAP=Mean arterial pressure.

**Supplementary Table S7: Relative Risks (RR) per standard deviation (SD) higher and 95% confidence intervals (CIs) for cognitive decline/dementia for different blood pressure (BP) parameters by stroke subtype**

| **BP Parameter** | **Ischemic stroke**  **RR (95% CI)** | **Not ischemic stroke**  **RR (95% CI)** | **Test for interaction** | **Hemorrhagic stroke**  **RR (95% CI)** | **Not hemorrhagic stroke**  **RR (95% CI)** | **Test for interaction** |
| --- | --- | --- | --- | --- | --- | --- |
| **SBP** |  |  |  |  |  |  |
| Baseline SBP | 1.00 (0.90-1.11) | 1.01 (0.89-1.15) | 0.87 | 0.99 (0.81-1.20) | 0.99 (0.91-1.09) | 0.96 |
| Mean SBP | 1.16 (1.01-1.32) | 1.22 (1.03-1.45) | 0.63 | 1.22 (0.9-1.62) | 1.17 (1.04-1.31) | 0.79 |
| Max SBP | 1.17 (1.03-1.32) | 1.15 (0.98-1.35) | 0.90 | 1.15 (0.87-1.51) | 1.16 (1.05-1.29) | 0.93 |
| SBP variability (SD) | 1.11 (1.01-1.21) | 1.00 (0.86-1.16) | 0.28 | 1.09 (0.86-1.39) | 1.07 (0.99-1.16) | 0.89 |
| SBP Load | 1.17 (1.03-1.32) | 1.14 (0.97-1.35) | 0.83 | 1.11 (0.85-1.46) | 1.16 (1.05-1.29) | 0.78 |
| SBP Slope | 1.11 (0.99-1.24) | 0.98 (0.85-1.15) | 0.24 | 1.05 (0.82-1.33) | 1.07 (0.97-1.18) | 0.88 |
| Last SBP | 1.16 (1.04-1.30) | 1.03 (0.89-1.19) | 0.24 | 1.14 (0.91-1.44) | 1.11 (1.01-1.23) | 0.86 |
| **DBP** |  |  |  |  |  |  |
| Baseline DBP | 0.98 (0.89-1.09) | 1.10 (0.96-1.26) | 0.25 | 1.05 (0.85-1.30) | 1.01 (0.93-1.10) | 0.78 |
| Mean DBP | 1.13 (0.98-1.30) | 1.27 (1.05-1.53) | 0.35 | 1.06 (0.77-1.46) | 1.18 (1.05-1.32) | 0.56 |
| Max DBP | 1.12 (0.98-1.26) | 1.31 (1.11-1.55) | 0.16 | 1.08 (0.83-1.41) | 1.18 (1.06-1.32) | 0.56 |
| DBP variability (SD) | 1.04 (0.94-1.14) | 1.16 (1.01-1.33) | 0.24 | 1.02 (0.85-1.23) | 1.08 (0.99-1.18) | 0.62 |
| DBP Load | 1.22 (1.08-1.37) | 1.26 (1.05-1.51) | 0.79 | 1.06 (0.82-1.38) | 1.24 (1.12-1.38) | 0.28 |
| DBP Slope | 1.16 (1.03-1.30) | 1.00 (0.84-1.18) | 0.17 | 0.86 (0.66-1.10) | 1.13 (1.02-1.25) | 0.08 |
| Last DBP | 1.17 (1.04-1.32) | 1.04 (0.88-1.22) | 0.25 | 1.04 (0.80-1.35) | 1.13 (1.01-1.25) | 0.58 |
| **PP** |  |  |  |  |  |  |
| Baseline PP | 1.01 (0.91-1.12) | 0.96 (0.83-1.10) | 0.56 | 0.94 (0.73-1.22) | 0.96 (0.83-1.10) | 0.77 |
| Mean PP | 1.11 (0.97-1.27) | 1.13 (0.95-1.35) | 0.87 | 1.27 (0.98-1.65) | 1.10 (0.98-1.24) | 0.43 |
| Max PP | 1.07 (0.94-1.22) | 1.08 (0.92-1.28) | 0.94 | 1.27 (0.97-1.67) | 1.06 (0.95-1.18) | 0.29 |
| PP variability (SD) | 1.05 (0.95-1.15) | 0.99 (0.86-1.14) | 0.52 | 1.17 (0.92-1.49) | 1.01 (0.93-1.10) | 0.26 |
| PP load | 1.11 (0.97-1.27) | 1.07 (0.89-1.28) | 0.75 | 1.25 (0.95-1.64) | 1.08 (0.96-1.21) | 0.40 |
| Last PP | 1.09 (0.97-1.22) | 1.02 (0.88-1.18) | 0.57 | 1.16 (0.93-1.45) | 1.06 (0.96-1.17) | 0.56 |
| **MAP** |  |  |  |  |  |  |
| Baseline MAP | 0.99 (0.89-1.10) | 1.06 (0.93-1.20) | 0.50 | 1.02 (0.84-1.23) | 1.00 (0.92-1.09) | 0.92 |
| Mean MAP | 1.16 (1.01-1.33) | 1.27 (1.06-1.51) | 0.46 | 1.16 (0.87-1.57) | 1.19 (1.06-1.33) | 0.89 |
| Max MAP | 1.17 (1.03-1.32) | 1.24 (1.06-1.46) | 0.59 | 1.13 (0.86-1.47) | 1.19 (1.08-1.32) | 0.71 |
| MAP variability (SD) | 1.09 (1.00-1.20) | 1.09 (0.95-1.26) | 0.97 | 1.07 (0.87-1.32) | 1.10 (1.01-1.19) | 0.85 |
| MAP load | 1.21 (1.07-1.36) | 1.21 (1.02-1.43) | 0.99 | 1.10 (0.85-1.42) | 1.21 (1.09-1.35) | 0.49 |
| Last MAP | 1.18 (1.05-1.33) | 1.04 (0.89-1.21) | 0.21 | 1.11 (0.87-1.42) | 1.13 (1.02-1.25) | 0.89 |

Note: p-value calculated using t-test between the BP parameter and stroke subtype was used to test the significance of the interaction term. The results were adjusted for age, sex, age at completion of highest education, region of residence, baseline BP measure, randomized BP treatment allocation, Mini-Mental State Examination (MMSE) score at baseline, body mass index (BMI), estimated glomerular filtration rate (eGFR), smoking, current drinking status (yes/no), and diabetes. SBP=Systolic blood pressure; DBP=Diastolic blood pressure; PP=Pulse pressure; MAP=Mean arterial pressure

**Supplementary Table S8: Relative risk (RR) per standard deviation (SD) higher and 95% confidence intervals (CIs) for cognitive decline/dementia for different blood pressure (BP) parameters by history of transient ischemic attack (TIA)**

| **BP Parameter** | **TIA**  **RR (95% CI)** | **No TIA**  **RR (95% CI)** | **Test for interaction** |
| --- | --- | --- | --- |
| **SBP** |  |  |  |
| Baseline SBP | 1.08 (0.92-1.26) | 0.98 (0.90-1.08) | 0.38 |
| Mean SBP | 1.22 (1.01-1.48) | 1.16 (1.02-1.32) | 0.68 |
| Max SBP | 1.16 (0.98-1.37) | 1.16 (1.04-1.30) | 0.97 |
| SBP variability | 0.96 (0.82-1.12) | 1.11 (1.02-1.22) | 0.15 |
| SBP Load | 1.19 (0.99-1.42) | 1.15 (1.02-1.29) | 0.79 |
| SBP Slope | 1.14 (0.98-1.33) | 1.04 (0.93-1.16) | 0.40 |
| Last SBP | 1.15 (0.98-1.35) | 1.10 (0.98-1.22) | 0.65 |
| **DBP** |  |  |  |
| Baseline DBP | 1.07 (0.91-1.26) | 1.01 (0.92-1.11) | 0.62 |
| Mean DBP | 1.22 (0.98-1.52) | 1.16 (1.02-1.32) | 0.73 |
| Max DBP | 1.18 (0.97-1.42) | 1.18 (1.05-1.33) | 0.96 |
| DBP variability | 1.03 (0.86-1.23) | 1.09 (1.00-1.20) | 0.57 |
| DBP Load | 1.22 (0.98-1.50) | 1.24 (1.11-1.39) | 0.87 |
| DBP Slope | 1.19 (0.97-1.45) | 1.09 (0.97-1.21) | 0.47 |
| Last DBP | 1.15 (0.94-1.42) | 1.12 (1.00-1.25) | 0.80 |
| **Pulse Pressure** |  |  |  |
| Baseline PP | 1.05 (0.89-1.23) | 0.97 (0.88-1.07) | 0.47 |
| Mean PP | 1.15 (0.95-1.40) | 1.10 (0.96-1.25) | 0.71 |
| Max PP | 1.08 (0.91-1.30) | 1.07 (0.95-1.21) | 0.92 |
| PP variability | 0.93 (0.80-1.09) | 1.06 (0.97-1.17) | 0.20 |
| PP load | 1.13 (0.92-1.37) | 1.08 (0.95-1.23) | 0.76 |
| Last PP | 1.11 (0.95-1.31) | 1.04 (0.93-1.16) | 0.57 |
| **MAP** |  |  |  |
| Baseline MAP | 1.08 (0.92-1.27) | 1.00 (0.91-1.09) | 0.42 |
| Mean MAP | 1.25 (1.03-1.53) | 1.18 (1.04-1.34) | 0.64 |
| Max MAP | 1.18 (0.99-1.42) | 1.19 (1.07-1.34) | 0.93 |
| MAP variability | 0.99 (0.84-1.18) | 1.13 (1.03-1.23) | 0.25 |
| MAP load | 1.23 (1.01-1.49) | 1.20 (1.07-1.34) | 0.84 |
| Last MAP | 1.17 (0.99-1.39) | 1.12 (1.00-1.25) | 0.69 |

Note: p-value calculated using t-test between the BP parameter and history of TIA was used to test the significance of the interaction term. The results were adjusted for age, sex, age at completion of highest education, region of residence, baseline BP measure, randomized BP treatment allocation, Mini-Mental State Examination (MMSE) score at baseline, body mass index (BMI), estimated glomerular filtration rate (eGFR), smoking, current drinking status (yes/no), and diabetes. SBP=Systolic blood pressure; DBP=Diastolic blood pressure; PP=Pulse pressure; MAP=Mean arterial pressure.

**Supplementary Table S9: Relative risk (RR) per standard deviation (SD) higher and 95% confidence intervals (CIs) for cognitive decline/dementia for different blood pressure (BP) parameters by history of having more than one cerebrovascular event or not**

| **BP Parameter** | **>1 previous cerebrovascular event** | **Not >1 previous cerebrovascular event** | **Test for interaction** |
| --- | --- | --- | --- |
| **SBP** |  |  |  |
| Baseline SBP | 1.03 (0.90-1.18) | 1.00 (0.90-1.10) | 0.73 |
| Mean SBP | 1.28 (1.08-1.54) | 1.14 (1.00-1.29) | 0.33 |
| Max SBP | 1.21 (1.03-1.41) | 1.13 (1.00-1.27) | 0.56 |
| SBP variability (SD) | 1.05 (0.90-1.22) | 1.07 (0.98-1.17) | 0.82 |
| SBP Load | 1.29 (1.08-1.53) | 1.11 (0.99-1.25) | 0.22 |
| SBP Slope | 1.16 (0.98-1.37) | 1.04 (0.93-1.16) | 0.32 |
| Last SBP | 1.17 (0.99-1.38) | 1.10 (0.98-1.22) | 0.56 |
| **DBP** |  |  |  |
| Baseline DBP | 1.02 (0.89-1.16) | 1.04 (0.94-1.14) | 0.84 |
| Mean DBP | 1.22 (1.01-1.48) | 1.16 (1.02-1.32) | 0.68 |
| Max DBP | 1.13 (0.96-1.32) | 1.19 (1.06-1.35) | 0.628 |
| DBP variability (SD) | 0.98 (0.85-1.13) | 1.10 (1.00-1.21) | 0.24 |
| DBP Load | 1.28 (1.07-1.52) | 1.21 (1.07-1.36) | 0.64 |
| DBP Slope | 1.13 (0.95-1.35) | 1.10 (0.98-1.23) | 0.82 |
| Last DBP | 1.15 (0.96-1.38) | 1.13 (1.00-1.26) | 0.87 |
| **Pulse Pressure** |  |  |  |
| Baseline PP | 1.02 (0.88-1.19) | 0.97 (0.88-1.07) | 0.58 |
| Mean PP | 1.23 (1.01-1.50) | 1.07 (0.94-1.22) | 0.30 |
| Max PP | 1.15 (0.96-1.38) | 1.04 (0.92-1.17) | 0.42 |
| PP variability (SD) | 1.00 (0.85-1.18) | 1.03 (0.94-1.13) | 0.80 |
| PP load | 1.23 (1.01-1.51) | 1.05 (0.92-1.19) | 0.21 |
| Last PP | 1.12 (0.94-1.34) | 1.04 (0.94-1.16) | 0.52 |
| **MAP** |  |  |  |
| Baseline MAP | 1.03 (0.90-1.17) | 1.02 (0.92-1.12) | 0.91 |
| Mean MAP | 1.28 (1.07-1.53) | 1.17 (1.02-1.33) | 0.45 |
| Max MAP | 1.20 (1.03-1.40) | 1.18 (1.04-1.32) | 0.85 |
| MAP variability (SD) | 1.04 (0.90-1.20) | 1.10 (1.00-1.21) | 0.55 |
| MAP load | 1.29 (1.10-1.52) | 1.17 (1.04-1.31) | 0.36 |
| Last MAP | 1.18 (0.99-1.39) | 1.12 (1.01-1.26) | 0.68 |

Note: p-value calculated using t-test between the BP parameter and having more than one previous cerebrovascular event was used to test the significance of the interaction term. The results were adjusted for age, sex, age at completion of highest education, region of residence, baseline BP measure, randomized BP treatment allocation, Mini-Mental State Examination (MMSE) score at baseline, body mass index (BMI), estimated glomerular filtration rate (eGFR), smoking, current drinking status (yes/no), and diabetes. SBP=Systolic blood pressure; DBP=Diastolic blood pressure; PP=Pulse pressure; MAP=Mean arterial pressure.

**Supplementary Table S10: Relative risk (RR) per standard deviation (SD) higher and 95% confidence intervals (CIs) for cognitive decline/dementia for different measures of blood pressure (BP) variability**

| **BP Parameter** | **Unadjusted** | **Basic adjusted** | **Multiple adjusted** |
| --- | --- | --- | --- |
| **SBP** | **RR (95% CI)** | **RR (95% CI)** | **RR (95% CI)** |
| SBP variability SD | 1.19 (1.11-1.28) | 1.10 (1.02-1.19) | 1.07 (0.99-1.16) |
| SBP variability CV | 1.15 (1.07-1.24) | 1.08 (1.00-1.16) | 1.05 (0.97-1.14) |
| **DBP** |  |  |  |
| DBP variability SD | 1.12 (1.04-1.21) | 1.09 (1.01-1.18) | 1.08 (1.00-1.17) |
| DBP variability CV | 1.13 (1.05-1.22) | 1.07 (0.99-1.16) | 1.06 (0.98-1.14) |
| **PP** |  |  |  |
| PP variability SD | 1.16 (1.08-1.25) | 1.05 (0.97-1.14) | 1.03 (0.95-1.11) |
| PP variability CV | 1.04 (0.96-1.12) | 1.02 (0.94-1.10) | 1.00 (0.93-1.08) |
| **MAP** |  |  |  |
| MAP variability SD | 1.17 (1.09-1.26) | 1.11 (1.03-1.20) | 1.09 (1.01-1.18) |
| MAP variability CV | 1.15 (1.07-1.24) | 1.09 (1.01-1.18) | 1.07 (0.99-1.16) |

Note: The results were adjusted for age, sex, age at completion of highest education, region of residence, baseline BP measure, randomized BP treatment allocation, Mini-Mental State Examination (MMSE) score at baseline, body mass index (BMI), estimated glomerular filtration rate (eGFR), smoking, current drinking status (yes/no), and diabetes. SBP=Systolic blood pressure; CV=Coefficient of variation; DBP=Diastolic blood pressure; PP=Pulse pressure; MAP=Mean arterial pressure.

**Supplementary Table S11: Relative risk (RR) per standard deviation (SD) higher and 95% confidence intervals (CIs) for cognitive decline/dementia for different measures of blood pressure (BP) variability adjusted by mean BP level**

| **BP Parameter** | **Multiple adjusted** | **Further adjustment by mean BP level** |
| --- | --- | --- |
|  | **RR (95% CI)** | **RR (95% CI)** |
| SBP variability SD | 1.07 (0.99-1.16) | 1.05 (0.97-1.14) |
| DBP variability SD | 1.08 (1.00-1.17) | 1.07 (0.99-1.16) |
| PP variability SD | 1.03 (0.95-1.11) | 1.01 (0.94-1.10) |
| MAP variability SD | 1.09 (1.01-1.18) | 1.08 (0.99-1.16) |

Note: The results were adjusted for age, sex, age at completion of highest education, region of residence, baseline BP measure, randomized BP treatment allocation, Mini-Mental State Examination (MMSE) score at baseline, body mass index (BMI), estimated glomerular filtration rate (eGFR), smoking, current drinking status (yes/no), and diabetes. SBP=Systolic blood pressure; DBP=Diastolic blood pressure; PP=Pulse pressure; MAP=Mean arterial pressure.

**Supplementary Table S12: Odds ratios (OR) per standard deviation (SD) higher and 95% confidence intervals (CIs) for cognitive decline/dementia for different blood pressure (BP) parameters by comparison of binomial and multinomial logistic regression**

| **BP Parameter** | **Binomial logistic regression model**  **OR 95% CI** | **Multinomial model**  **OR 95% CI** |
| --- | --- | --- |
| **SBP** |  |  |
| Baseline SBP | 1.00 (0.91-1.10) | 1.01 (0.92-1.12) |
| Mean SBP | 1.23 (1.08-1.39) | 1.24 (1.10-1.41) |
| Max SBP | 1.20 (1.06-1.35) | 1.22 (1.09-1.38) |
| SBP variability (SD) | 1.09 (0.99-1.19) | 1.10 (1.00-1.21) |
| SBP variability (CV) | 1.06 (0.97-1.16) | 1.08 (0.98-1.18) |
| SBP Load | 1.20 (1.07-1.35) | 1.23 (1.09-1.38) |
| SBP Slope | 1.08 (0.97-1.20) | 1.09 (0.98-1.21) |
| Last SBP | 1.14 (1.03-1.27) | 1.15 (1.03-1.28) |
| **DBP** |  |  |
| Baseline DBP | 1.04 (0.94-1.15) | 1.04 (0.95-1.15) |
| Mean DBP | 1.22 (1.07-1.38) | 1.23 (1.08-1.39) |
| Max DBP | 1.22 (1.08-1.37) | 1.23 (1.09-1.39) |
| DBP variability (SD) | 1.09 (1.00-1.20) | 1.10 (1.00-1.21) |
| DBP variability (CV) | 1.07 (0.97-1.17) | 1.08 (0.98-1.18) |
| DBP Load | 1.29 (1.15-1.45) | 1.31 (1.16-1.47) |
| DBP Slope | 1.13 (1.01-1.26) | 1.13 (1.01-1.26) |
| Last DBP | 1.16 (1.04-1.29) | 1.16 (1.04-1.30) |
| **PP** |  |  |
| Baseline PP | 0.98 (0.88-1.08) | 0.99 (0.89-1.09) |
| Mean PP | 1.14 (1.00-1.30) | 1.16 (1.01-1.32) |
| Max PP | 1.10 (0.97-1.24) | 1.11 (0.98-1.27) |
| PP variability (SD) | 1.03 (0.94-1.14) | 1.04 (0.95-1.15) |
| PP variability (CV) | 1.00 (0.91-1.10) | 1.01 (0.92-1.11) |
| Last PP | 1.08 (0.96-1.20) | 1.09 (0.97-1.22) |
| **MAP** |  |  |
| Baseline MAP | 1.02 (0.93-1.12) | 1.03 (0.94-1.13) |
| Mean MAP | 1.25 (1.10-1.41) | 1.26 (1.11-1.43) |
| Max MAP | 1.23 (1.10-1.38) | 1.25 (1.11-1.41) |
| MAP variability (SD) | 1.11 (1.01-1.22) | 1.13 (1.02-1.24) |
| MAP variability (CV) | 1.09 (0.99-1.19) | 1.10 (1.00-1.21) |
| Last MAP | 1.17 (1.05-1.30) | 1.17 (1.05-1.31) |

Note: SBP=Systolic blood pressure; CV=Coefficient of variation; DBP=Diastolic blood pressure; PP=Pulse pressure; MAP=Mean arterial pressure. Both models were adjusted for age, sex, age at completion of highest education, region of residence, randomized BP treatment allocation, Mini-Mental State Examination (MMSE) score at baseline, baseline BP, body mass index (BMI), estimated glomerular filtration rate (eGFR), smoking, current drinker or not, and diabetes.

**Supplementary Figures**

**Supplementary Figure S1: Timeline showing measurement occasions of the exposure and outcome period.** Note: BP=Blood pressure; MMSE=Mini-Mental State Examination.

**Supplementary Figure S2: Flowchart of participant selection for analysis.** Note: BP=Blood pressure; MMSE=Mini-Mental State Examination.

**Supplementary Figure S3: Splines of each diastolic blood pressure (DBP) parameter with the outcomes of cognitive decline/dementia.** Note: The results were adjusted for age, sex, age at completion of highest education, region of residence, baseline blood pressure (BP) measure, randomized BP treatment allocation, Mini-Mental State Examination (MMSE) score at baseline, body mass index (BMI), estimated glomerular filtration rate (eGFR), smoking, current drinking status (yes/no), and diabetes. RR=Relative Risk; CI=Confidence interval; SD=Standard deviation.

**Supplementary Figure S4: Splines of each mean arterial pressure (MAP) parameter with the outcomes of cognitive decline/dementia.** Note: The results were adjusted for age, sex, age at completion of highest education, region of residence, baseline blood pressure (BP) measure, randomized BP treatment allocation, Mini-Mental State Examination (MMSE) score at baseline, body mass index (BMI), estimated glomerular filtration rate (eGFR), smoking, current drinking status (yes/no), and diabetes. Note: RR=Relative risk; CI=Confidence interval; SD=Standard deviation.

**Supplementary Figure S5: Splines of each pulse pressure (PP) parameter with the outcomes of cognitive decline/dementia.** Note: The results were adjusted for age, sex, age at completion of highest education, region of residence, baseline blood pressure (BP) measure, randomized BP treatment allocation, Mini-Mental State Examination (MMSE) score at baseline, body mass index (BMI), estimated glomerular filtration rate (eGFR), smoking, current drinking status (yes/no), and diabetes. Note: RR=Relative Risk; CI=Confidence interval; SD=Standard deviation.

**Supplementary discussion**

A. **Directions for future research**

Future studies should consider using additional instruments to the MMSE to detect MCI and smaller changes in cognitive decline. For example, the Montreal Cognitive Assessment (MoCA) has been found to have higher sensitivity and specificity for detecting MCI in comparison to the MMSE.^7^ Trials with antihypertensive drugs have focused largely on cardiovascular outcomes, with cognitive decline or dementia as secondary outcomes. Future studies could benefit from prioritizing cognitive decline/dementia as the primary outcomes, with adequate years of follow-up. Finally, BPV calculated using home blood pressure or 24-hour ambulatory monitoring, could provide valuable prognostic information for cognitive decline/dementia risk compared to that of the conventional single office BP measurement alone.^8^

**References**

1.	Tzourio C, Anderson C, Chapman N, Woodward M, Neal B, MacMahon S, Chalmers J. Effects of blood pressure lowering with perindopril and indapamide therapy on dementia and cognitive decline in patients with cerebrovascular disease. *Arch Intern Med*. 2003;163:1069–1075. doi: 10.1001/archinte.163.9.1069

2.	Bell CC. DSM-IV: Diagnostic and Statistical Manual of Mental Disorders. *JAMA*. 1994;272:828–829. doi: 10.1001/jama.1994.03520100096046

3.	Papaioannou TG, Protogerou AD, Vrachatis D, Konstantonis G, Aissopou E, Argyris A, Nasothimiou E, Gialafos EJ, Karamanou M, Tousoulis D, et al. Mean arterial pressure values calculated using seven different methods and their associations with target organ deterioration in a single-center study of 1878 individuals. *Hypertens Res*. 2016;39:640–647. doi: 10.1038/hr.2016.41

4.	Wang N, Harris K, Hamet P, Harrap S, Mancia G, Poulter N, Williams B, Zoungas S, Woodward M, Chalmers J, et al. Cumulative Systolic Blood Pressure Load and Cardiovascular Risk in Patients With Diabetes. *Journal of the American College of Cardiology*. 2022;80:1147–1155. doi: 10.1016/j.jacc.2022.06.039

5.	Grams ME, Sang Y, Ballew SH, Matsushita K, Astor BC, Carrero JJ, Chang AR, Inker LA, Kenealy T, Kovesdy CP, et al. Evaluating Glomerular Filtration Rate Slope as a Surrogate End Point for ESKD in Clinical Trials: An Individual Participant Meta-Analysis of Observational Data. *J Am Soc Nephrol*. 2019;30:1746–1755. doi: 10.1681/asn.2019010008

6.	Maeda T, Woodward M, Jun M, Sakamoto Y, Chen X, Matsushita K, Mancia G, Arima H, Anderson CS, Chalmers J, et al. Risk of recurrent stroke and dementia following acute stroke by changes in kidney function: results from the Perindopril Protection Against Recurrent Stroke Study. *J Hypertens*. 2024;42:1313–1321. doi: 10.1097/hjh.0000000000003711

7.	Nasreddine ZS, Phillips NA, Bédirian V, Charbonneau S, Whitehead V, Collin I, Cummings JL, Chertkow H. The Montreal Cognitive Assessment, MoCA: a brief screening tool for mild cognitive impairment. *J Am Geriatr Soc*. 2005;53:695–699. doi: 10.1111/j.1532-5415.2005.53221.x

8.	Schutte AE, Kollias A, Stergiou GS. Blood pressure and its variability: classic and novel measurement techniques. *Nature Reviews Cardiology*. 2022;19:643–654. doi: 10.1038/s41569-022-00690-0
